# A Wnt-related gene expression signature to improve the prediction of prognosis and tumor microenvironment in gastric cancer

**DOI:** 10.3389/fgene.2022.1035099

**Published:** 2022-12-06

**Authors:** Shuai Kong, Zhi Li, Yuanyuan Wang, Zheming Zhang, Xianghao Jia, Xinxin Gao, Bicong Cong, Fangxu Zhang, Jing Zhang, Chunning Zheng

**Affiliations:** ^1^ Gastrointestinal Surgery, Shandong Provincial Hospital, Jinan, China; ^2^ Department of Pharmacy, The Fourth People’s Hospital of Jinan, Jinan, China; ^3^ Department of Oncology, The Second Affiliated Hospital of Shandong University of Traditional Chinese Medicine, Jinan, China; ^4^ General Surgery, Weifang Medical University, Weifang, China; ^5^ General Foreign Major, Shandong First Medical University, Tai’an, China; ^6^ Gastrointestinal Surgery, Shandong First Medical University, Jinan, China; ^7^ General Surgery, The Fourth People’s Hospital of Jinan, Jinan, China; ^8^ Department of Oncology, HaploX Biotechnology, Shenzhen, China

**Keywords:** Wnt signaling pathway, mutation, tumor microenvironment, prognosis, gastric cancer

## Abstract

**Background:** Most gastric cancer (GC) patients were diagnosed in the advanced stages without obvious symptoms, which resulted in the increased risk of death. Although the combination therapies have showed survival benefit of patients, there is still urgent need to explore the underlying mechanisms of GC development and potential novel targets for clinical applications. Numerous studies have reported the upregulation of Wnt signaling pathway in human GC, which play important role during GC development and progression. However, the current understanding of Wnt signaling pathway is still limited due to its complexity and contradictory effect on different stages of GC tumor microenvironment.

**Method:** We used The Cancer Genome Atlas (TCGA) and Gene Expression Omnibus (GEO) dataset to screen Wnt signaling pathway-associated genes by ssGSEA and correlation analysis. Three molecular subtypes were constructed based on a consistent clustering analysis. The key Wnt-related genes were screened through univariate cox analysis, lasso, and stepwise regression. In addition, the Gene Set Enrichment Analysis (GSEA) were performed to explore potential molecular pathways regulated by the Wnt-related gene signatures. ESTIMATE was utilized for evaluating the immune cell populations in GC tumor microenvironment.

**Results:** Three molecular subtypes associated to Wnt were identified, and 7 key Wnt-related genes were screened to establish a predictive RiskScore model. These three molecular subtypes showed significant prognostic differences and distinct functional signaling pathways. We also found the downregulated immune checkpoint expression in the clust1 with good prognosis. The RiskScore model was successfully validated in GSE26942 dataset. Nomogram based on RiskScore and Gender had better prognostic predictive ability.

**Conclusion:** In summary, our study showed that the Wnt-related genes could be used to predict prognosis of GC patients. The risk model we established showed high accuracy and survival prediction capability.

## Introduction

Gastric cancer (GC) is the major cause of cancer-related mortality worldwide (Takechi et al., 2020), which is induced by genetic predisposition and environmental factors (González et al., 2002). According to the previous reports, the main risk factor for most GC patients is *Helicobacter pylori* infection (Conteduca et al., 2013). Other factors such as Epstein-Barr virus infection, geographical location, smoking, and abnormal diet were also reported to be associated with GC development and progression (Shokal and Sharma, 2012; Correale and Gaitan, 2015; Spence et al., 2017). Early diagnostic rate of GC has been improved by the use of gastroscopy (Eusebi et al., 2020). In the early stage of GC, gastrectomy is the prioritized strategy for the radical cure of patients. However, most patients with GC progressing to advanced stages without obvious symptoms, which results in the increased risk of death. Although the combination treatments have showed survival benefit of patients, it is still urgent to explore the underlying mechanisms of GC development and potential novel targets for clinical applications.

The Wnt signaling pathway functions most commonly in biological processes including embryonic development and self-renewal of tissues (Yang et al., 2016; Gavert and Ben-Ze’ev, 2007). Specifically, it is a complex signaling pathway related to multiple downstream channels activated upon the binding of Wnt ligands to its membrane receptor (Komiya and Habas, 2008). Abundant preclinical and clinical studies have demonstrated that the Wnt signaling pathway could progress the malignant transformation, tumor progression, and resistance to conventional cancer treatments (Sullivan et al., 2010; Anastas and Moon, 2013; Galluzzi et al., 2019). Growing evidence indicates that aberrant Wnt signaling may also induce immunosuppressive signals in the tumor microenvironment, thereby promoting resistance to various anti-cancer therapies including immune checkpoint blockade therapy (Galluzzi et al., 2019; Zhou et al., 2022). Numerous studies have reported the upregulation of Wnt signaling pathway in human GC due to the oncogenetic mutation or overexpression of Wnt ligand and its receptors, which linked alterations of Wnt signaling to GC development and progression (Yang et al., 2018; Nie et al., 2022). Although great progress has been made in exploring the mechanism of this pathway for the treatment and prediction of GC, the current understanding of Wnt signaling pathway is still limited due to its complexity and contradictory effect on different stages of GC tumor microenvironment.

Herein, in this study, we collected GC patient samples from The Cancer Genome Atlas (TCGA) dataset and screened Wnt signaling pathway-associated genes by single sample gene set enrichment analysis (ssGSEA) and correlation analysis. Three gene-related molecular subtypes were constructed to explore their functions in GC tumor immune microenvironment by analyzing different immune cell scores. The 7 key Wnt-related genes were screened through univariate cox analysis, lasso, and stepwise regression. Then, we established a stable predictive RiskScore model for clinical outcome. The model was further improved by the decision tree model, which showed high prediction accuracy and survival prediction capability.

## Materials and methods

### Data collection and sources

The mutation data, copy number variation data, and RNA-seq data for GC patients were downloaded through the TCGA GDC API. We then removed the samples without survival time and survival status. The expression profile data and survival data of the GSE26942 dataset were downloaded from NCBI’s Gene Expression Omnibus (GEO) official website (https://www.ncbi.nlm.nih.gov/geo/).

The KEGG_WNT_SIGNALING_PATHWAY data was downloaded from the Molecular Signatures Database (https://www.gsea-msigdb.org/gsea/index.jsp) to obtain gene information of the related pathways.

### RNA-seq data preprocessing

For the TCGA RNA-seq data, we first removed samples without clinical follow-up information such as the loss of survival time and status. After screening, a total of 333 primary tumor samples were included. Then, the ensemble was converted to gene symbol and the average expression was taken when multiple probes correspond to a gene name We then took base 2 logarithm of the expression file of fragments per kilobase of transcript per million fragments mapped (FPKM) for further analysis.

For the GEO data, we removed normal tissue samples and the samples without clinical follow-up information and ensured that the survival time of all samples is greater than 0. 93 tumor tissues and 25,127 genes were finally obtained. Then, the probes were converted into gene symbols through the platform annotation file. We also removed the mean of multiple gene names corresponding to one probe. The average expression was taken when multiple probes correspond to a gene name.

### Construction of molecular subtypes of related genes

Consensus clustering was used to construct a consistency matrix and cluster the samples (Wilkerson and Hayes, 2010). Using the expression data of Wnt-related genes, the molecular subtypes of the samples were obtained. The “pam” algorithm and “pearson” were utilized as the metric distance and we performed 500 bootstraps. Each bootstrap process included 80% of the training set patients. The number of clusters was set from 2 to 10, and the optimal classification was determined by calculating the cumulative distribution function (CDF) to obtain the molecular subtypes of the sample.

### Risk model

We first identified the genes associated with differences among the subtypes and selected differentially expressed genes with significant prognosis (|LogFC|>1; FDR<0.05). Further, the number of genes was reduced by lasso regression to obtain phenotype-related prognostic genes. We then calculated the risk score for each patient using the following formula: RiskScore = Σβi×Expi, where Expi refers to the gene expression level of the phenotypic prognosis-related genes, and β is the lasso Cox regression coefficient of the corresponding gene. After zscore for risk score, and according to the threshold “0,” the patients were divided into RiskScore high and low risk groups. Kaplan-Meier method was utilized for prognostic analysis and the log-rank test was used to evaluate the significant difference.

### Gene set enrichment analysis

We performed GSEA to investigate signaling pathways regulated by the different molecular subtypes by using all candidate gene sets in the HALLMARK database (Liberzon et al., 2015). The Gene Ontology (GO) and Kyoto Encyclopedia of Genes and Genomes (KEGG) enrichment analysis on the 2,443 genes were performed by the WebGestaltR package (Liao et al., 2019; Wang et al., 2021a). The *p* values smaller than 0.05 was determined as statistically significant. The correlation coefficients were also calculated by R package.

### Calculation of immune cell abundance in tumor microenvironment

The characteristic genes of 28 immune cells were obtained from the previous study (Charoentong et al., 2017) and the scores of these immune cells were calculated by using the ssGSEA algorithm (Finotello and Trajanoski, 2018). At the same time, we also used the ESTIMATE software to calculate the proportion of immune cells between low- and high-risk groups (Luo et al., 2020; Fan et al., 2021).

### Decision tree

Recursive partitioning analysis was performed to construct a survival decision tree for risk stratification with R package “rpart”.

## Results

### Screening of genes related to Wnt signaling pathway in GC

We first calculated the correlation score of Wnt signaling pathway from each patient based on the 151 relevant genes. Here, we screened a total of 2,443 genes that were associated with the Wnt correlation score (cor>0.4 and *p* < 0.001). Next, GO and KEGG enrichment analysis were performed on the 2,443 genes. For GO functional annotations, 960 of which were annotated with significant differences in biological process (BP) (Supplementary Figure S1A). There are 169 genes annotated with significant differences in cellular component (CC) and 116 genes were annotated with significant differences in molecular function (MF) (Supplementary Figures S1B, C). Additionally, 73 genes were observed with significant differences in KEGG enrichment analysis (Supplementary Figure S1D). Among these genes, the top signaling pathways were most related to the extracellular structure, extracellular matrix (ECM), and ECM-receptor interaction.

### Construction of molecular subtypes based on genes correlated with Wnt score

**FIGURE 1:**
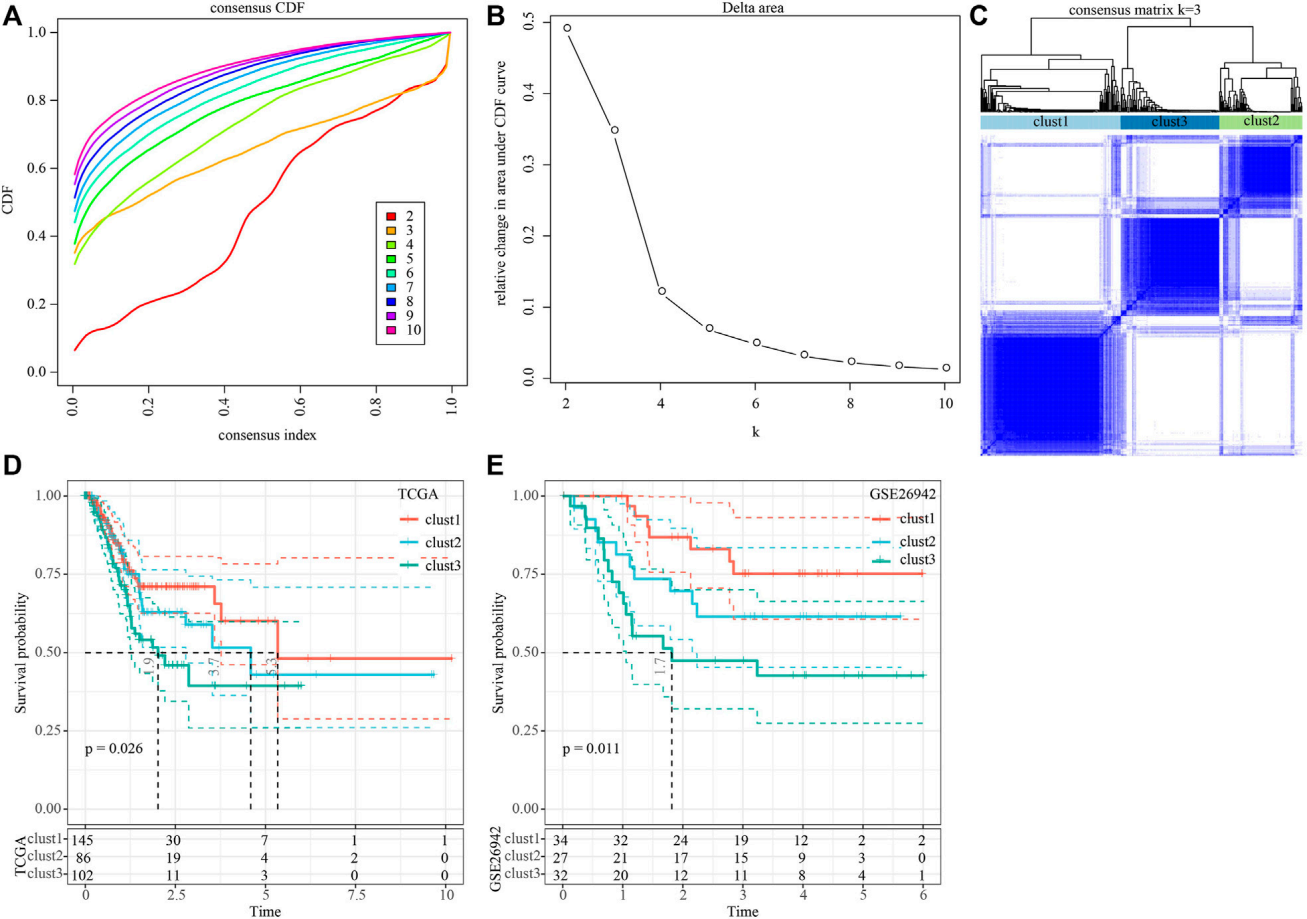
Construction of molecular subtypes based on genes positively correlated with Wnt score. **(A)** CDF curve of TCGA cohort sample; **(B)** CDF Delta area curve of TCGA cohort sample. The horizontal axis represents the category number k and the vertical axis represents the relative change in area under CDF curve; **(C)** Sample clustering heat map when consensus k = 3; **(D)** KM curve of the relationship between the prognosis of three subtypes in the TCGA cohort; **(E)** KM curves for the prognosis of the three subtypes in the GSE26942 cohort.

We then performed univariate cox analysis on the 2,443 genes correlated with the Wnt score and found that a total of 41 genes were highly related to prognosis, of which 0 genes were protective genes (Protect, HR < 1), and 41 were risk genes (Risk, HR > 1). We aggregated the positively correlated genes by consensus class on TCGA data and determined the optimal number of clusters and observed the CDF Delta area curve. As shown in Figures 1A,B, when cluster was selected as 3, it showed a relatively stable clustering result. Finally, we chose k = 3 to obtain three molecular subtypes (Figure 1C). Further prognosis analysis of these three molecular subtypes showed significant prognostic differences (Figure 1D). Compared to clust2 and clust3, clust1 showed the best survival benefit. In addition, we performed molecular typing on the GSE26942 data by using the same method and the significant differences in the prognosis of different molecular types were observed (Figure 1E), which was consistent with the TCGA dataset.

**FIGURE 2:**
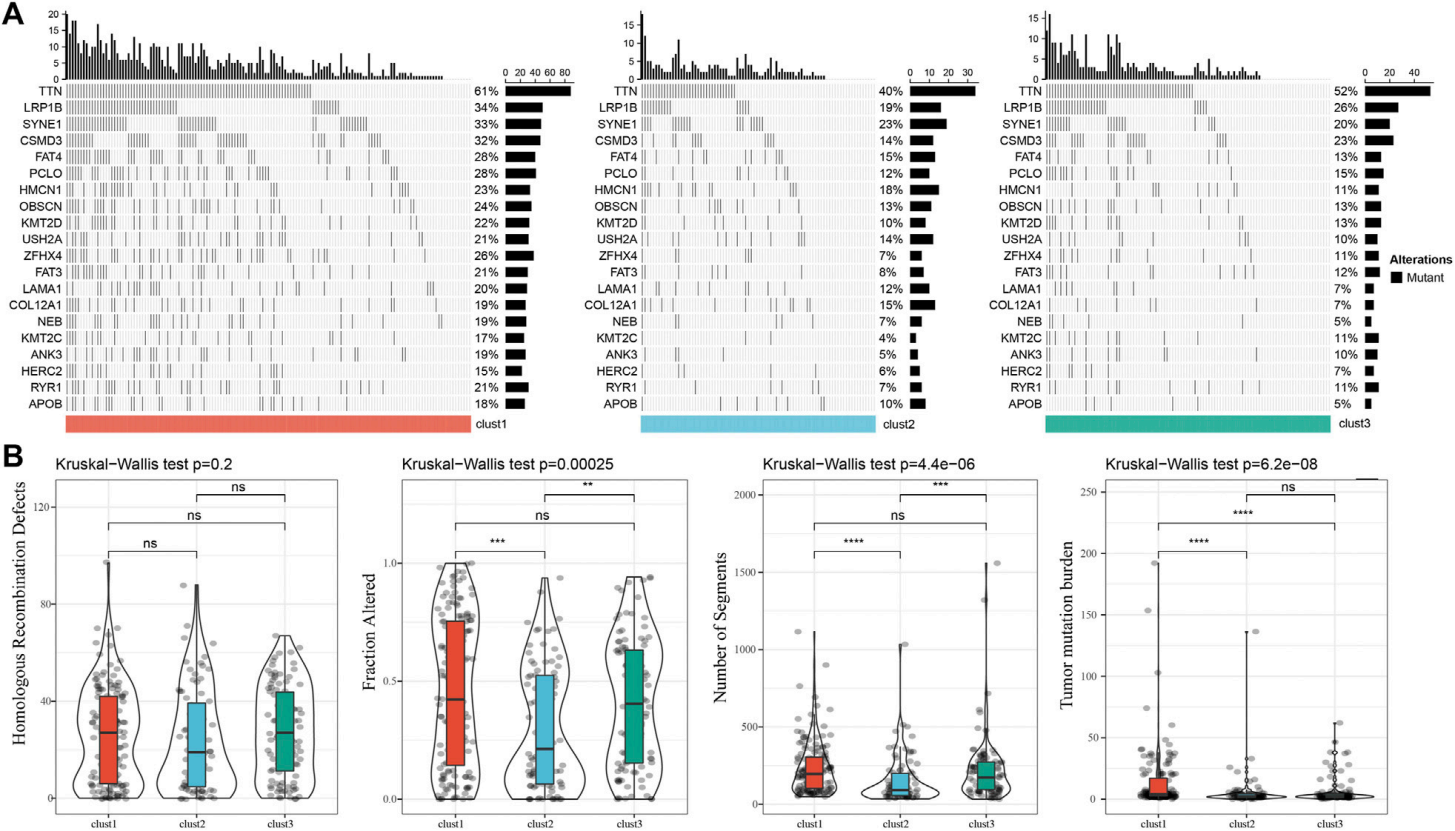
Genomic alterations in molecular subtypes of the TCGA cohort. **(A)** Somatic mutation analysis of different molecular subtypes in the TCGA cohort (Fisher’s exact test); **(B)** Comparison of Homologous Recombination Defects, Fraction Altered, Number of Segments and Tumor mutation burden among different molecular subtypes in the TCGA cohort.

We further analyzed the differences in clinicopathological characteristics among different molecular subtypes in the TCGA cohort and compared their distribution of different clinical characteristics. As shown in Supplementary Figure S2 the significant differences were found in T stage, Stage, and patient survival status among the three subtypes. We also explored differences in genomic alterations among different molecular subtypes. The mutation characteristics of the top 20 genes in each subtype were shown in Figure 2A. We compared the distribution of Homologous Recombination Defects, Fraction Altered, Number of Segments, and tumor mutation burden between subtypes. Compared to clust2 and clust3, clust1 showed significant increase of Fraction Altered, Number of Segments, and tumor mutation burden (Figure 2B).

### Pathway analysis and immune characterization of molecular subtypes

**FIGURE 3:**
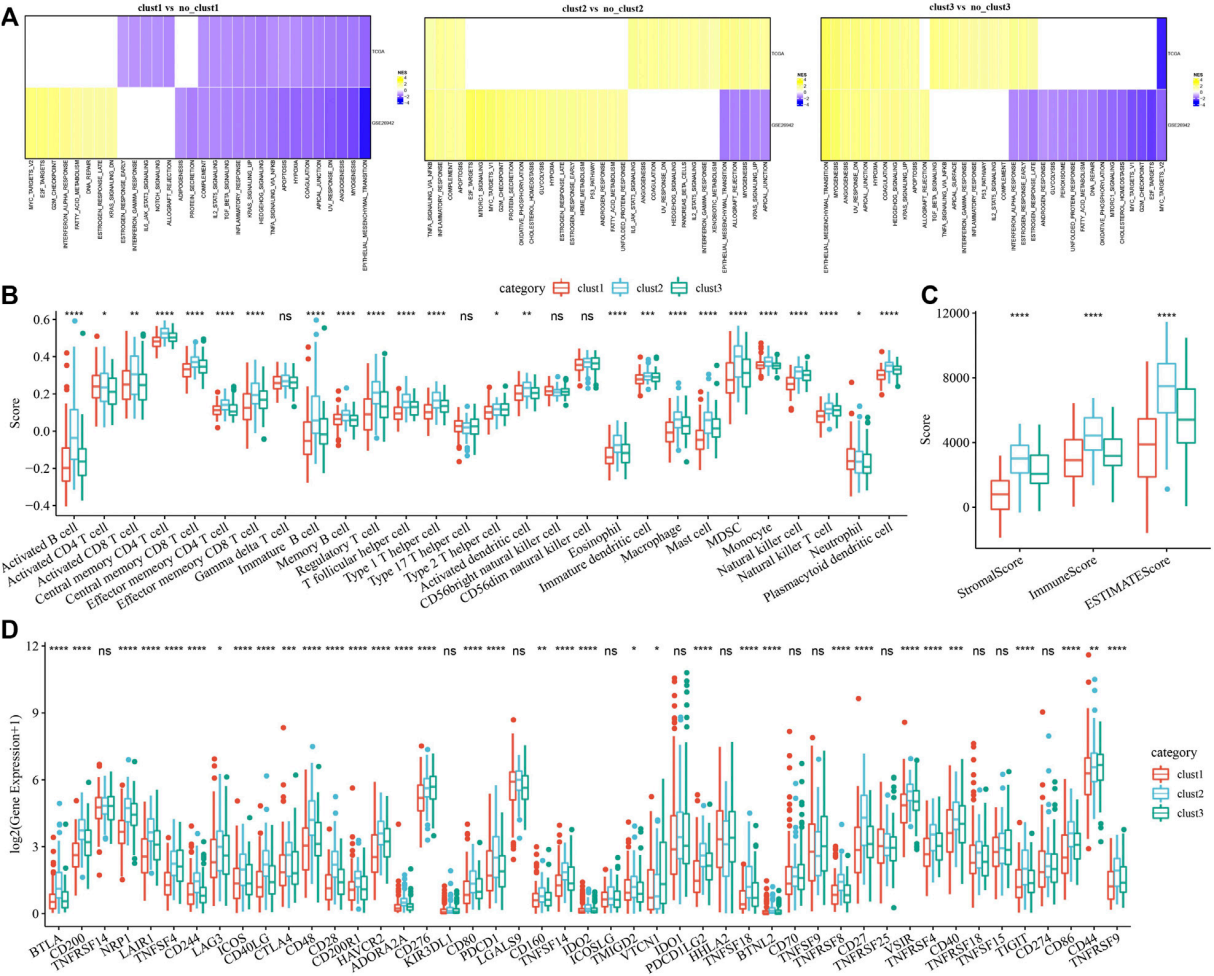
Pathway analysis and immune characterization of molecular subtypes. **(A)** A heatmap demonstrating normalized enrichment scores (NESs) of Hallmark pathways calculated by comparing clust1 with clust2 (with a false discovery rate (FDR) < 0.05); **(B)** Comparison of 28 immune cell scores between different subtypes; **(C)** Comparison of immune scores in different subtypes; **(D)** Comparison of immune checkpoint genes between different subtypes. Kruskal. Test, *p < 0.05; **p < 0.01; ***p < 0.001; and ****p < 0.0001.

To investigate pathways of different biological processes among these three subtypes, we performed the GSEA enrichment analysis. As shown in Figure 3A, clust1 had 20 inhibited pathways in the TCGA cohort, while 17 inhibited pathways were found in the GSE26942 cohort. For clust2, no inhibited pathways were observed in the TCGA cohort, and 5 pathways were inhibited in the GSE26942 cohort. Compared with no_clust3, 1 pathway was inhibited in clust3 in the TCGA cohort, and 16 pathways were inhibited in the GSE26942 cohort. To further elucidate differences in the tumor immune microenvironment of GC patients between different molecular subtypes, we assessed the extent of immune cell infiltration in TCGA cohort by using the expression levels of genes in immune cells. As shown in Figure 3B, we found significant differences in some immune cells such as CD4 T, CD8 T, natural killer (NK) cells, macrophages and MDSCs among these three subtypes. The immune score was also evaluated by the ESTIMATE method (Figure 3C). However, the immune score of clust1 with good prognosis was lower than that of the clust2 and clust3 subtypes, which might be induced by other immunosuppressive factors such as ECM that could form the second physical barrier and attenuate the penetration of immune cells inside the tumor tissue.

We therefore examined the expression of immune checkpoint genes in the three subtypes. Compared to clust2 and clust3, we found that most of the immune checkpoint genes were sharply downregulated in clust1 (Figure 3D), indicating that immune checkpoint inhibition mainly contributed to good prognosis of clust1. Our findings indicated that the prognosis of GC patients among the tree Wnt-related molecular subtypes was highly associated with tumor immune microenvironment and their related signaling pathways.

### Identification of key genes and construction of Wnt-related risk model

**FIGURE 4:**
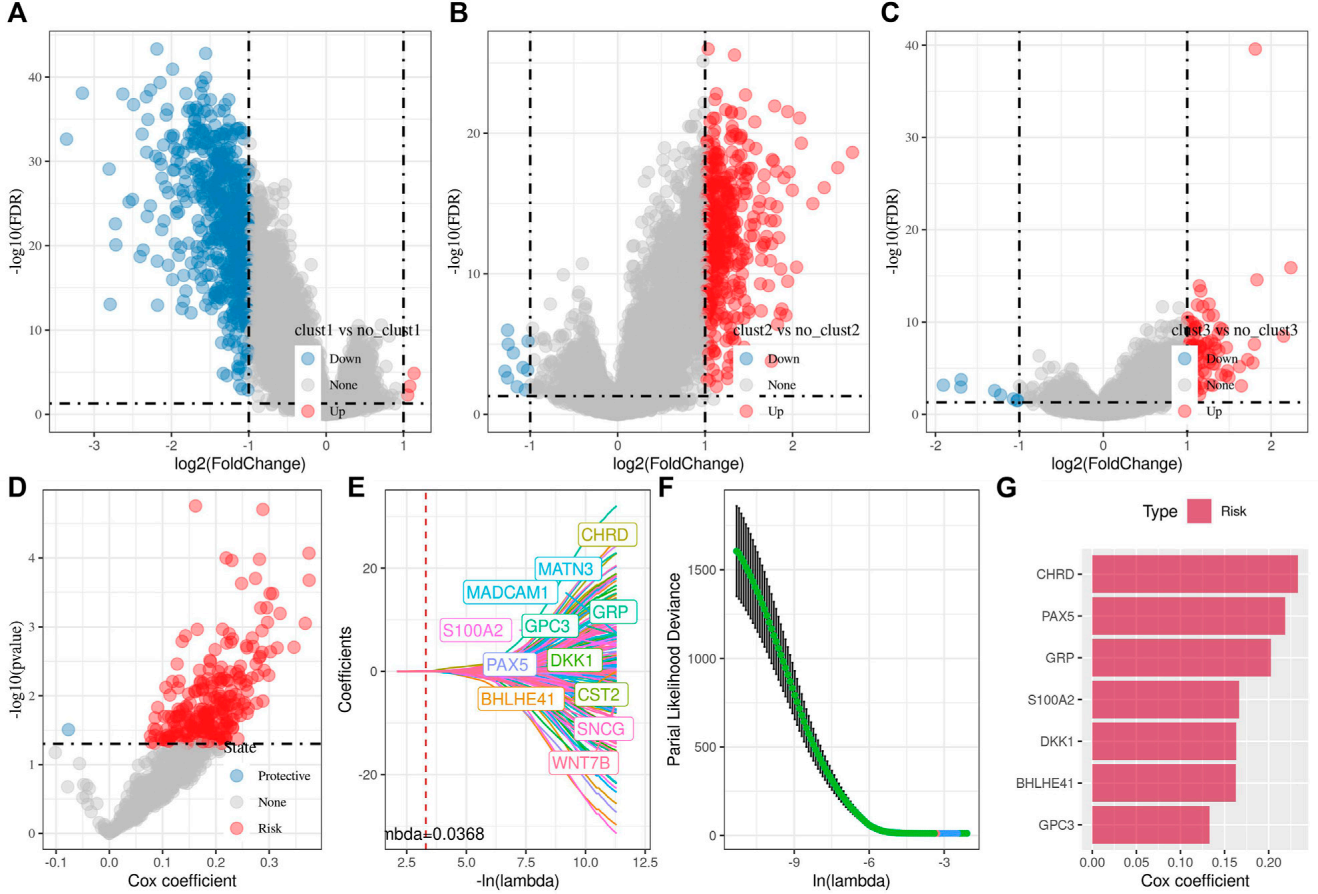
Differential analysis among three subtypes for the identification of key genes. **(A)** Volcano plot of differential analysis of clust1 vs. no_clust1 in TCGA cohort; **(B)** Volcano plot of differential analysis of clust2 vs. no_clust2 in TCGA cohort; **(C)** Volcano plot of differential analysis of clust3 vs. no_clust3 in TCGA cohort; **(D)** A total of 774 promising candidates were identified among the DEGs; **(E)** Trajectory of each independent variable with lambda; **(F)** Confidence interval under lambda; **(G)** Multivariate cox analysis, coefficients of prognostic-related genes.

In the previous analysis, we identified three distinct molecular subtypes through the Wnt signaling pathway score-related genes, and found differences between the subtypes through clinical phenotype, mutation, immune signature, and pathway analysis. Then, we performed differential analysis on clust1 vs. no_clust1 subtypes, clust2 vs. no_clust2, clust3 vs. no_clust3 subtypes to screen differential genes. In clust1 vs. no_clust1, we screened 379 up-regulated genes and 603 downregulated genes, while 441 upregulated genes and 10 downregulated genes in clust2 vs. no_clust2. There were 84 genes with upregulated expression and 8 genes with downregulated expression in clust3 vs. no_clust3. The volcano plots of difference analysis were shown in Figures 4A–C. We finally screened a total of 773 differential genes for further analysis. Next, we performed univariate cox analysis on the 773 differential genes and screened 259 genes related to prognosis. As shown in Figure 4D, there were 258 risk genes and 1 protective gene. To reduce the number of genes, these 259 genes were further compressed by using lasso regression for the risk model construction. The change trajectory of each independent variable was analyzed as shown in Figure 4E, from which with the gradual increase of lambda, the coefficient of the independent variable tends to 0. The penalty parameter was established through 10-fold cross validation to build the model and analyze the confidence interval under each lambda (Figure 4F). The model tended to be optimal when lambda was 0.0368. We therefore selected 12 genes as the target genes and used stepwise multivariate regression analysis. As shown in Figure 4G, 7 genes including CHRD, BHLHE41, GRP, GPC3, PAX5, S100A2, and DKK1 were identified as correlated genes affecting prognosis. The formula was as follows:

RiskScore=0.233*CHRD+0.163*BHLHE41+0.202*GRP+0.133*GPC3+0.219*PAX5+0.167*S100A2+0.163*DKK1

**FIGURE 5:**
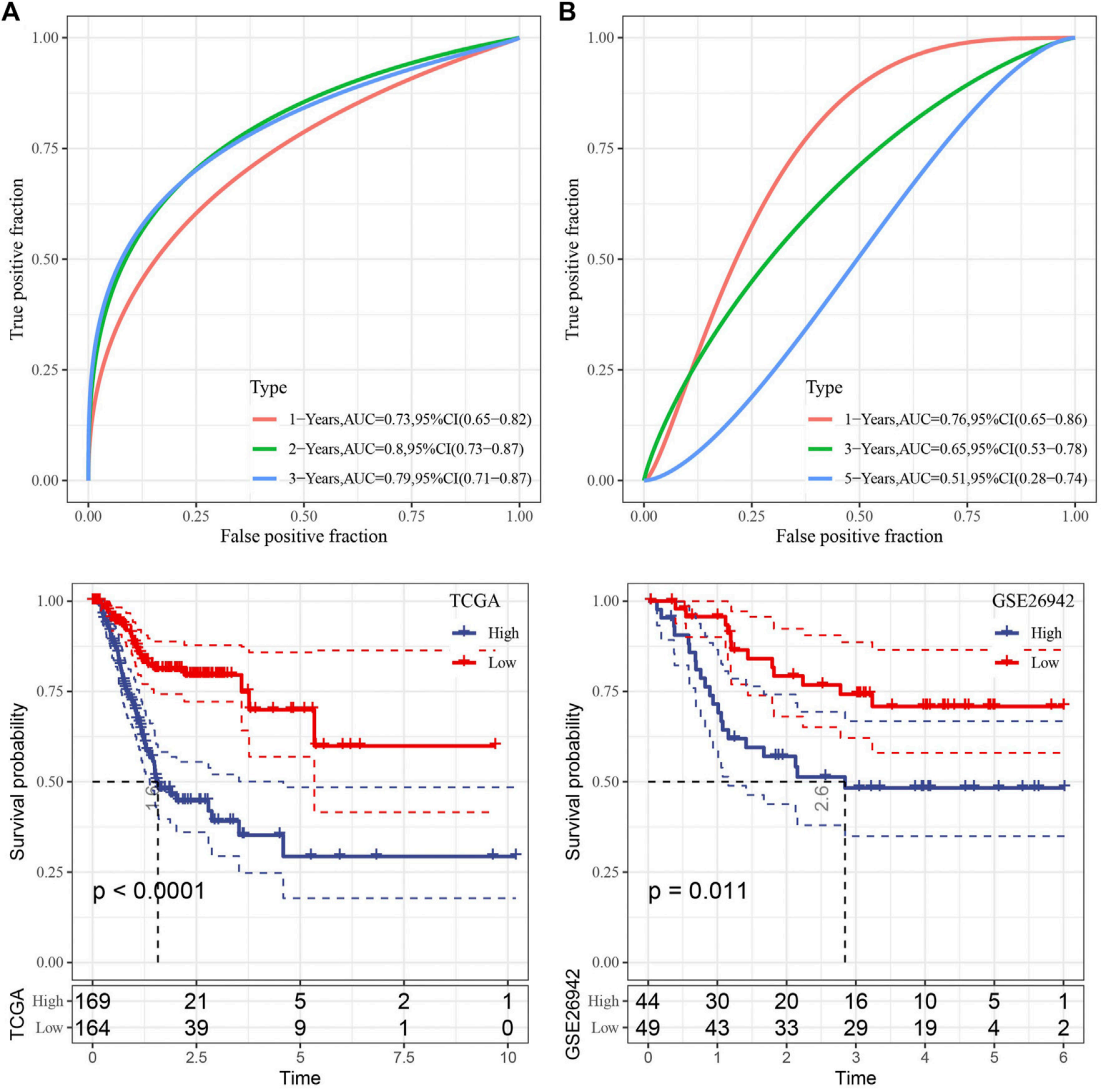
Risk model construction. **(A)** ROC curve and KM curve of risk model constructed by 7 genes in TCGA dataset; **(B)** ROC curve and KM curve of risk model constructed by 7 genes in GSE26942 dataset.

We then used the TCGA data as the training data set and calculated the RiskScore of each sample through the 7 gene expression levels. The receiver operation characteristic (ROC) curve analysis of the prognostic classification on the RiskScore were performed and analyzed. As shown in Figure 5A, the prognostic prediction classification efficiency was analyzed in 1, 2, and 3 years, respectively, of which the area under the time-dependent ROC curves (AUC) reached 0.7 in 1–3 years, indicating the predictive ability of this model.

Furthermore, zscore was also performed on the RiskScore and the samples were divided into high-risk group when the Riskscore greater than zero and those with less than zero as low-risk group. High-risk group showed poor survival benefit compared to low-risk group (Figure 5A). To better verify the robustness of the model, we used the GSE26942 dataset for validation and the risk model established was applied to perform prognostic classification on RiskScore. Similar results were obtained compared with that under TCGA dataset, indicating excellent predictive capability of this model (Figure 5B).

**FIGURE 6:**
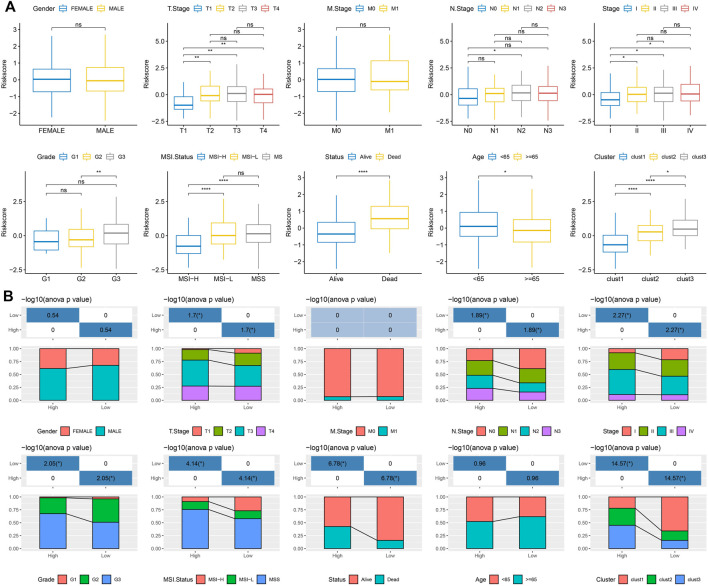
The relationship between the RiskScore and tumor clinical characteristics. **(A)** Differences in RiskScore of different phenotypes in the TCGA cohort (Wilcox. Test, *p < 0.05; **p < 0.01; ***p < 0.001; and ****p < 0.0001); **(B)** Comparison of clinical phenotypes between RiskScore groups in the TCGA cohort.

To examine the relationship between the RiskScore and tumor clinical characteristics, we analyzed the differences in RiskScore between different clinical phenotypes in the TCGA dataset. The results showed that the risk score increased with deepening of the clinical grade (Figure 6A). We also compared the differences in clinicopathological characteristics between the RiskScore groups in the TCGA cohort and found similar results (Figure 6B), indicating the good performance of the model to predict the clinical stage of GC progression.

### Mutation characteristics of high- and low-risk groups

We further explored differences in genomic alterations between high- and low-risk groups in the TCGA cohort. We screened out 9,922 genes and used the fisher test to screen for significant high-frequency mutations in each subtype. Finally, 1892 genes were obtained and the mutation characteristics of the top 20 genes in each subtype were shown in Supplementary Figure S3. In addition, we compared the distribution of Homologous Recombination Defects, Fraction Altered, Number of Segments, and tumor mutation burden between subtypes. Compared to low-risk group, the Homologous Recombination Defects increased, while tumor mutation burden sharply decreased in the high-risk group (Supplementary Figure S3B), which was consistent with the observations in the clust1 with best prognosis. These results indicated that the risk model we established could predict the clinical outcome according to the mutation burden in GC patients.

### Pathway characteristics between two risk groups

**FIGURE 7:**
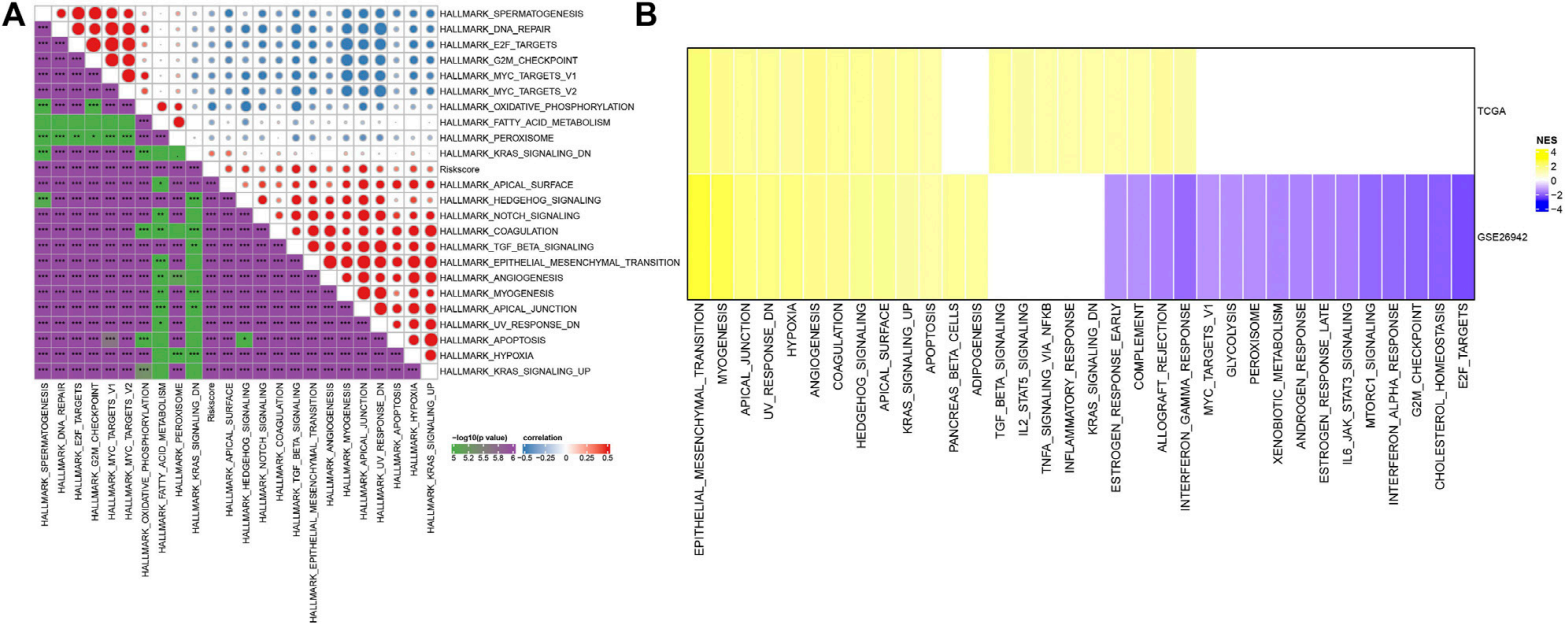
Pathway characteristics between RiskScore grouping. **(A)** Correlation analysis between HALLMARK pathways with RiskScore correlations greater than 0.3 and RiskScore; **(B)** A heatmap demonstrating normalized enrichment scores (NESs) of Hallmark pathways calculated by comparing High with Low (with a false discovery rate (FDR) < 0.05).

To observe the relationship between the RiskScore of different samples and their biological functions, gene expression profiles corresponding to the tumor samples were selected in the TCGA cohort and calculated the scores of each sample on different biological functions. The ssGSEA score of each function corresponding to each sample was obtained, and the correlation between these functions and RiskScore was further calculated. As shown in Figure 7A, the function with a correlation greater than 0.3 was selected, from which the RiskScore showed a positive correlation between these pathways and GC samples. We next analyzed the differentially enriched pathways in GC samples. As shown in Figure 7B, 20 pathways were activated, and no inhibited pathways were observed in the TCGA cohort. Additionally, 13 activated pathways and 16 inhibited pathways were found in the GSE26942 cohort. Particularly, the pro-tumor signals including KRAS, TGF-β, and hypoxia pathways showed significant positive relationship with GC progression. These results suggested that RiskScore was related to the biological functions and tumor-enriched signaling pathways. The seven genes in the model might be involved in signaling pathway regulation and the RiskScore had biological support for predicting prognosis.

### RiskScore combined with clinicopathological features to further improve prognostic model and survival prediction

**FIGURE 8:**
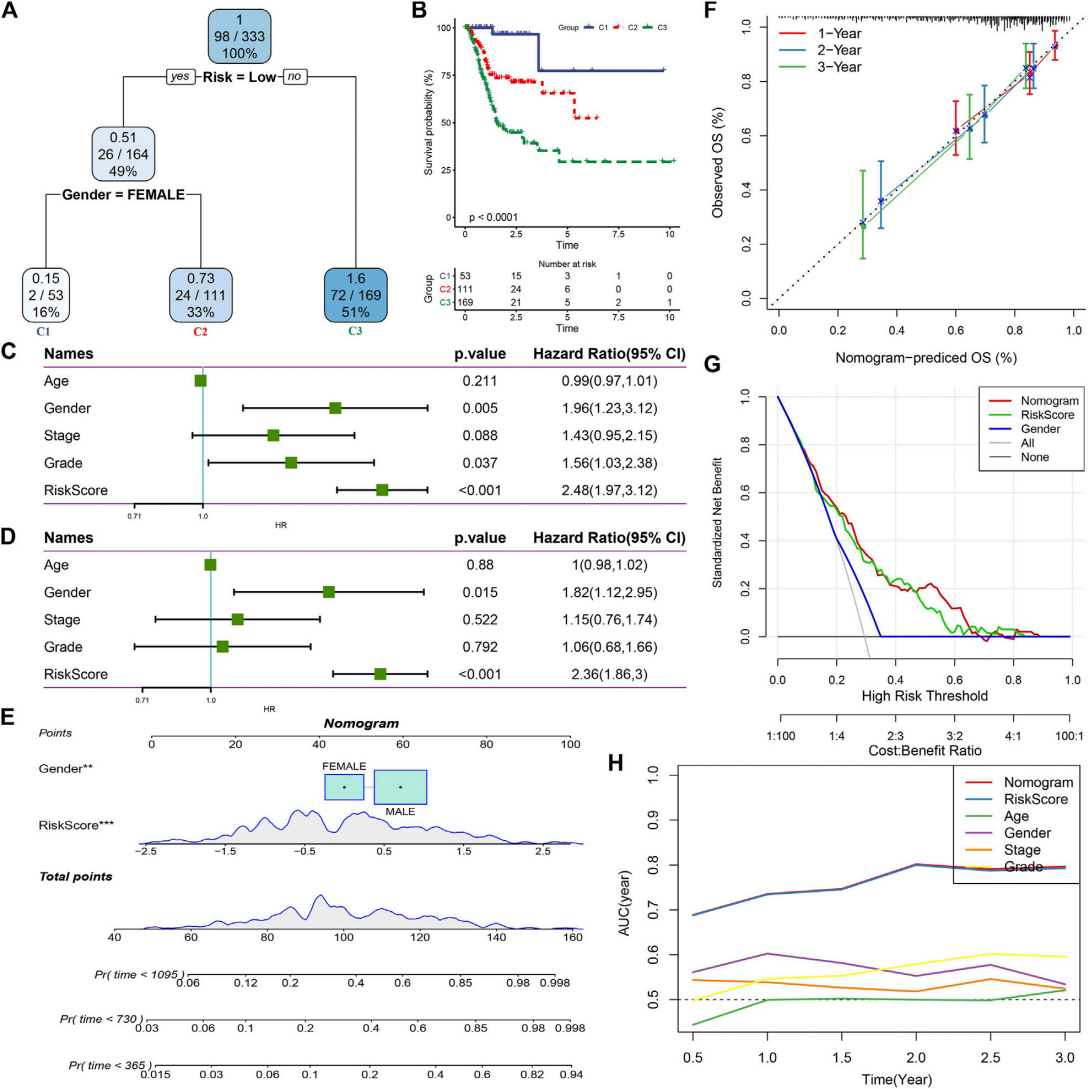
RiskScore combined with clinicopathological features to further improve prognostic model and survival prediction. **(A)** Patients with full-scale annotations including RiskScore, stage, gender and age were used to build a survival decision tree to optimize risk stratification; **(B)** Significant differences of overall survival were observed among the three risk subgroup; **(C)** Univariate cox analysis of RiskScore and clinical characteristics; **(D)** Multivariate Cox analysis of RiskScore and clinical characteristics; **(E)** Nomogram model; **(F)** Calibration curves for 1, 2, and 3 years of nomogram; **(G)** Nomogram decision curve; **(H)** Compared with other clinicopathological features, the nomogram exhibited the most powerful capacity for survival prediction.

Here, we constructed a decision tree based on patient age, gender, stage, grade, and RiskScore in the TCGA cohort. The results showed that only RiskScore and gender remained in the decision tree, and three distinct risk subgroups were identified (Figure 8A). Among them, gender and RiskScore were the most powerful parameters. There were significant differences in overall survival between the three risk subgroups (Figure 8B). We found RiskScore as the most significant prognostic factor by univariate and multivariate Cox regression analysis of RiskScore and clinical characteristics (Figures 8C,D). To verify the risk assessment and survival benefit of patients, we combined RiskScore and other clinicopathological features to build a nomogram as shown in Figure 8E. From the model results, RiskScore showed the greatest influence on the prediction of survival rate. Further, we evaluated the prediction accuracy of the model by using the calibration curve (Figure 8F). The predicted curves from the calibration points in 1, 2, and 3 years were nearly coincident with the standard curve, suggesting that the nomogram showed a good predictive performance. Moreover, decision curve analysis (DCA) was also used to evaluate the reliability of the risk model (Figure 8G). Both RiskScore and nomogram had significantly higher benefits than extreme curves. Moreover, compared with other clinicopathological features, nomogram and RiskScore showed the strongest survival predictive capability (Figure 8H).

## Discussion

Numerous studies have proved that various factors such as genetic predisposition and environmental factors were associated with GC development (González et al., 2002; Shokal and Sharma, 2012; Conteduca et al., 2013; Correale and Gaitan, 2015; Spence et al., 2017). Although improved diagnosis and prioritized gastrectomy strategy for early stage of GC patients have been achieved during the past decades, most GC patients were diagnosed to advanced stages without obvious symptoms, which induced poor prognosis. The preclinical and clinical studies have demonstrated that the Wnt signaling pathway could progress the GC malignant transformation, progression, and resistance to conventional cancer treatments (Sullivan et al., 2010; Anastas and Moon, 2013; Galluzzi et al., 2019). Growing evidence demonstrated the upregulation of Wnt signaling pathway in human GC (Yang et al., 2018; Nie et al., 2022). Although great progress has been made in exploring the mechanism of the Wnt pathways for the treatment and prediction of GC, the current understanding of this pathway is still limited. The next-generation sequencing data analysis from human patient samples has been demonstrated as a powerful tool to explore the mechanisms of cancer development and progression, which could execute the predictive risk model establishment for clinical outcome. In this study, we used RNA-seq data generated from GC patient samples to screen Wnt signaling pathway-associated genes. Then, we constructed three gene-related molecular subtypes to explore their functions in GC tumor immune microenvironment and established a risk prediction model for clinical applications.

Herein, we screened a total of 2,443 genes that were positively associated with Wnt signaling pathways. Most top Wnt-related genes were found to be responsible for ECM construction and remodeling to induce the tumor immunosuppressive microenvironment, which was consistent with previous reports (Sathe et al., 2020; Liu et al., 2021). We constructed three molecular subtypes and found that compared to clust2 and clust3, clust1 showed the best prognosis. However, the immune score and the levels of some immune cells such as CD4 T, CD8 T, and NK cells in clust1 were lower than those in clust2 and clust3. As reported, the T cell and NK cell infiltration inside the tumor microenvironment will drive the antitumor immunity by inducing the innate and adaptive immune response (Wang et al., 2018; Hu et al., 2019). The low level of immune cell infiltration in clust1 might result from other immunosuppressive factors such as fibroblast and M2-polarized macrophage that could form the second physical barrier and inhibit the penetration of immune cells inside the tumor tissue (Wang et al., 2021b). We also evaluated the expression level of immune checkpoint genes in the three subtypes. Interestingly, compared to clust2 and clust3, most of the immune checkpoint genes were significantly suppressed in clust1. These findings suggested that immune checkpoint inhibition instead of immune cell infiltration contributed to the good prognosis of clust1. Moreover, we found that Fraction Altered, Number of Segments, and tumor mutation burden had significance in three subtypes, which may also be responsible for the prognosis difference of three subtypes.

By screening of gene signatures, we identified seven significant genes including CHRD, BHLHE41, GRP, GPC3, PAX5, S100A2, and DKK1 as correlated genes with GC development. Among these genes, dickkopf-1 (DKK1) was reported as a secretory glycoprotein that can inhibit the activation of Wnt singling pathway, which should be considered as a therapeutic target and further explore its function in antitumor immunity (Liu et al., 2016; Jiang et al., 2021). Some studies have found paired box gene 5 (PAX5) promoter methylation in GC cells and tumor tissues that was significantly associated with the survival of GC patients, which is consistent with our findings of PAX5 and its related Wnt signaling pathway in GC (Otani et al., 2013; Deng et al., 2014). The correlation between these selected genes and GC prognosis might provide potential targets for the GC treatment.

We further established a prognostic risk model for clinical outcome prediction and performed validation studies. Collectively, the model we established has been evaluated that showed high accuracy and survival prediction capability. The findings here provide a potential future research direction in the effect of Wnt signaling pathway on GC development and migration. Additionally, the comprehensive Wnt signaling pathway in the various immune cell types and ECM-related cells in the tumor microenvironment could be further explored for the clinical diagnosis and treatment of GC patients.

## Conclusion

In this study, we screened Wnt signaling pathway-related genes by ssGSEA and correlation analysis from GC patient samples. Three molecular subtypes related to prognosis were constructed by Wnt-related genes and analyzed their function and immune-related pathways in GC. The 7 key Wnt-related genes were screened through univariate cox analysis, lasso, and stepwise regression. Then, we established the RiskScore clinical prognostic model, which is robust and independent of clinicopathological features, and has stable predictive performance in independent datasets. Finally, the prognostic model and survival prediction were further improved by the decision tree model, which showed high prediction accuracy and survival prediction capability.

## Contribution to the field statement

Three gene-related molecular subtypes were constructed, and 7 key Wnt-related genes were screened to establish a predictive RiskScore model. These three molecular subtypes showed significant prognostic differences and distinct functional signaling pathways. We also found the downregulated immune checkpoint expression in the clust1 with good prognosis.

## Data Availability

The original contributions presented in the study are included in the article/Supplementary Material, further inquiries can be directed to the corresponding author.
