# Supplementary figures and images for: A Wnt-related gene expression signature to improve the prediction of prognosis and tumor microenvironment in gastric cancer
Source: Front Genet. 2022 Dec 6;13:1035099. doi: 10.3389/fgene.2022.1035099 (PMC9763457; doi:10.3389/fgene.2022.1035099)

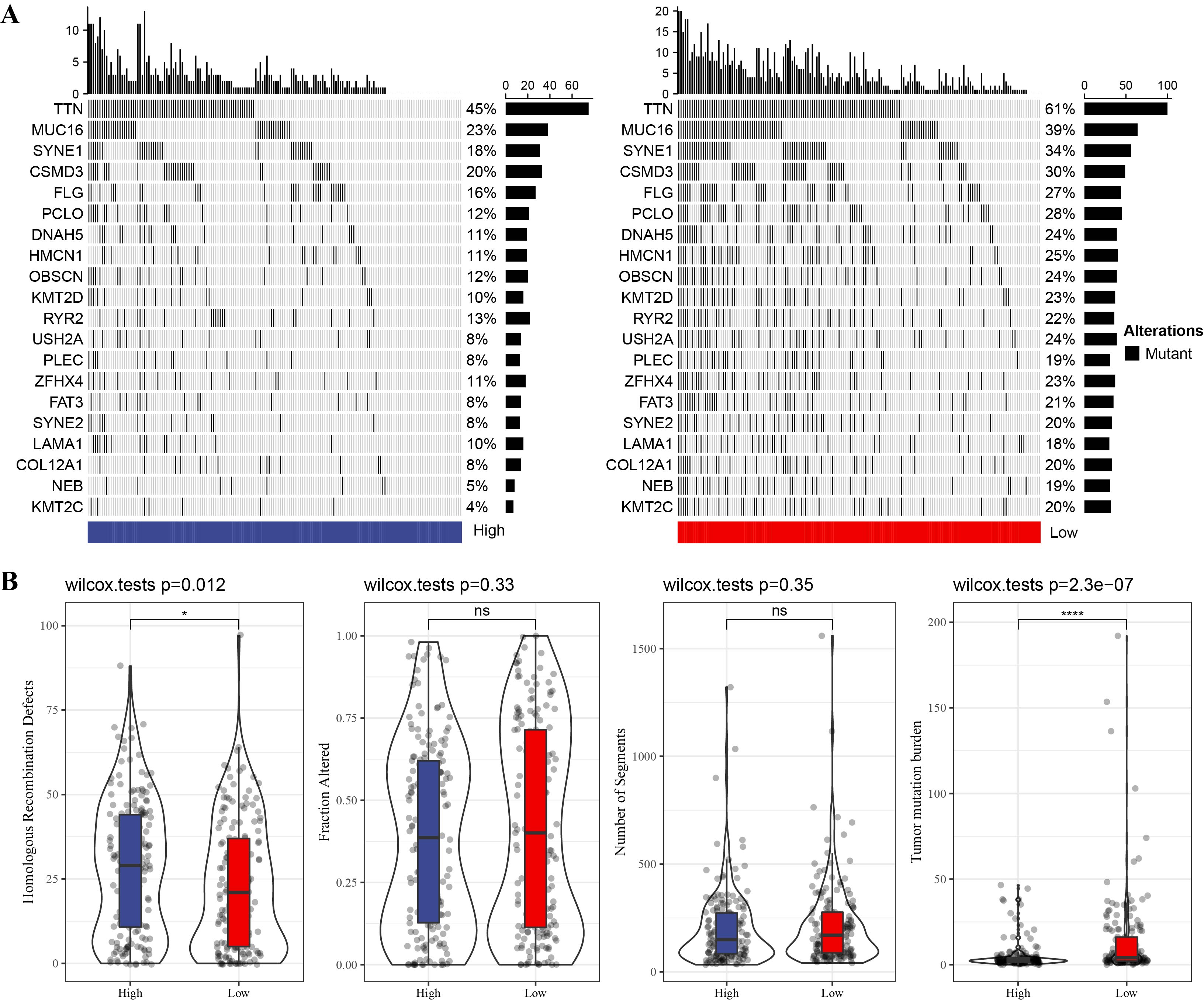

Supplement: Supplementary file 1 [file Image3.JPEG]

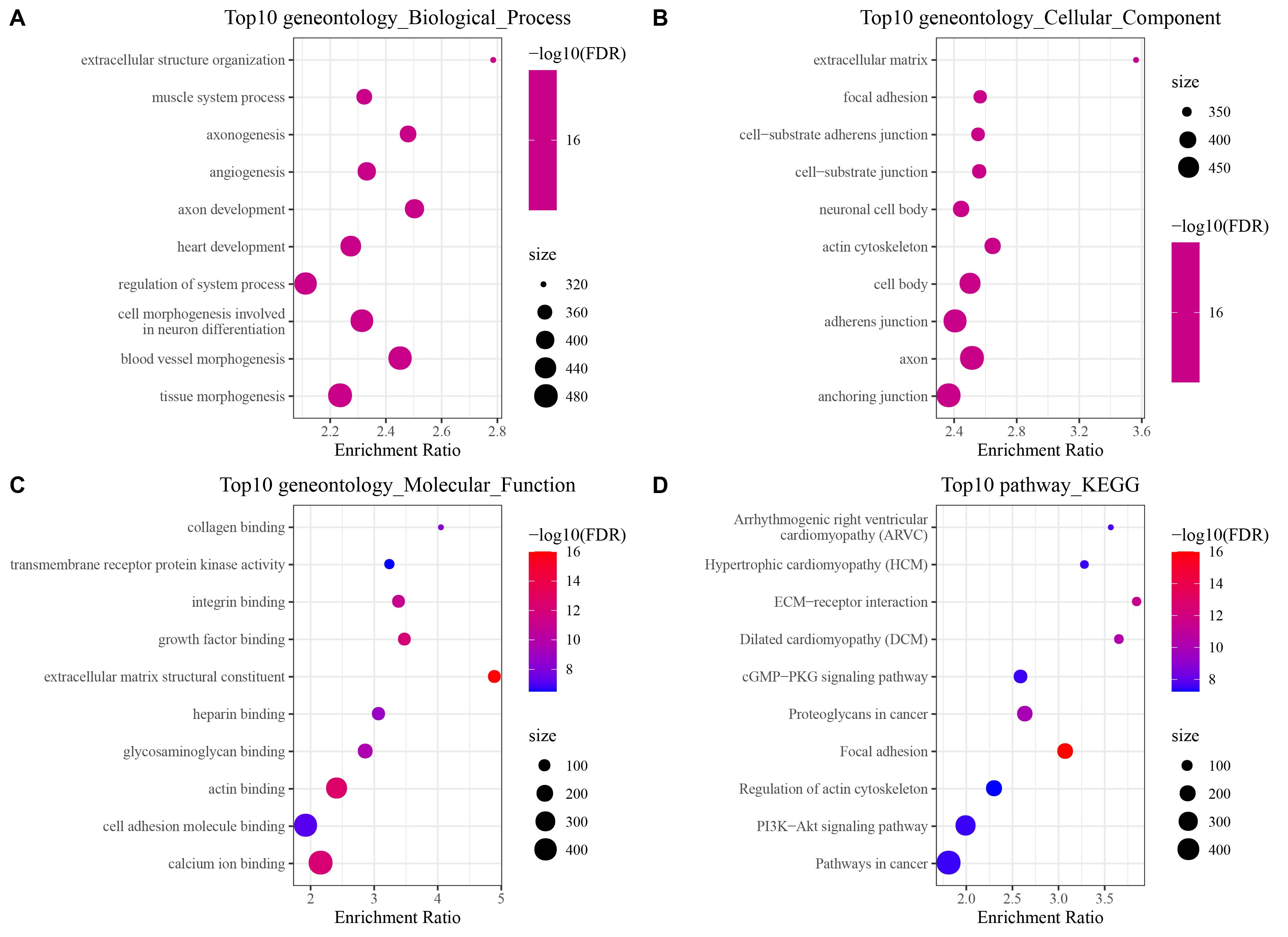

Supplement: Supplementary file 3 [file Image1.JPEG]

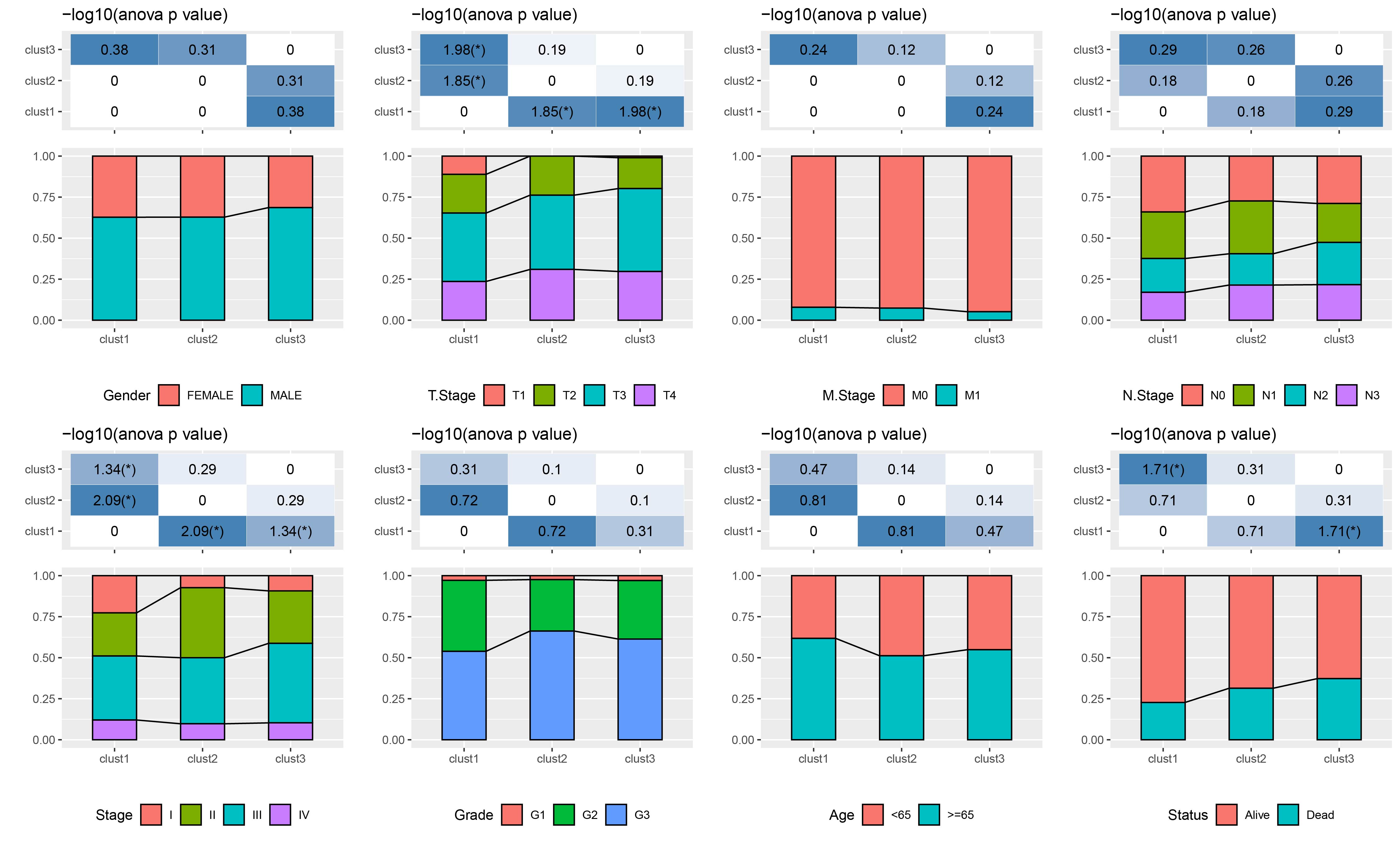

Supplement: Supplementary file 4 [file Image2.JPEG]
