# Supplementary material for: A Wnt-related gene expression signature to improve the prediction of prognosis and tumor microenvironment in gastric cancer
Source: Front Genet. 2022 Dec 6;13:1035099. doi: 10.3389/fgene.2022.1035099 (PMC9763457; doi:10.3389/fgene.2022.1035099)
Supplement: Supplementary file 2 [file Table1.DOCX]

Description of supplementary materials

Figure S1. Functional analysis of WNT-related genes. A: Biological process (BP) annotation map of genes positively correlated with WNT Score; B: Cellular component (CC) annotation map of genes positively correlated with WNT Score; C: Molecular function (MF) annotation map of genes positively correlated with WNT Score; D: KEGG annotation map of genes positively correlated with WNT Score.

Figure S2. Clinical information distribution of molecular subtypes in the TCGA cohort.

Figure S3. Mutation characteristics of high- and low- risk groups. Genomic alterations in molecular subtypes of the TCGA cohort. A: Somatic mutation analysis of high and low risk groups in the TCGA cohort (Fisher's exact test); B: Comparison of Homologous Recombination Defects, Fraction Altered, Number of Segments and Tumor mutation burden between high and low risk groups in the TCGA cohort.
