# Supplementary material for: A Wnt-related gene expression signature to improve the prediction of prognosis and tumor microenvironment in gastric cancer
Source: Front Genet. 2022 Dec 6;13:1035099. doi: 10.3389/fgene.2022.1035099 (PMC9763457; doi:10.3389/fgene.2022.1035099)
Supplement: Supplementary file 5 [file Table2.DOCX]

Data analyzed in this study please see:

<https://www.jianguoyun.com/p/DfcKaeIQharcChi8vNQEIAA>
